# Evolutionary Position and Leaf Toughness Control Chemical Transformation of Litter, and Drought Reinforces This Control: Evidence from a Common Garden Experiment across 48 Species

**DOI:** 10.1371/journal.pone.0143140

**Published:** 2015-11-17

**Authors:** Xu Pan, Yao-Bin Song, Can Jiang, Guo-Fang Liu, Xue-Hua Ye, Xiu-Fang Xie, Yu-Kun Hu, Wei-Wei Zhao, Lijuan Cui, Johannes H. C. Cornelissen, Ming Dong, Andreas Prinzing

**Affiliations:** 1 Key Laboratory of Hangzhou City for Ecosystem Protection and Restoration, College of Life and Environmental Sciences, Hangzhou Normal University, Hangzhou, China; 2 Institute of Wetland Research, Chinese Academy of Forestry, Beijing, China; 3 State Key Laboratory of Vegetation and Environmental Change, Institute of Botany, Chinese Academy of Sciences, Beijing, China; 4 Systems Ecology, Department of Ecological Science, Faculty of Earth and Life Sciences, VU University Amsterdam, 1081 HV Amsterdam, The Netherlands; 5 Université de Rennes 1, Centre National de la Recherche Scientifique Campus de Beaulieu, Research Unit Ecobio, Bâtiment 14 A, 35042 Rennes, France; Helmholtz Centre for Environmental Research (UFZ), GERMANY

## Abstract

Plant leaf litter is an important source of soil chemicals that are essential for the ecosystem and changes in leaf litter chemical traits during decomposition will determine the availability of multiple chemical elements recycling in the ecosystem. However, it is unclear whether the changes in litter chemical traits during decomposition and their similarities across species can be predicted, respectively, using other leaf traits or using the phylogenetic relatedness of the litter species. Here we examined the fragmentation levels, mass losses, and the changes of 10 litter chemical traits during 1-yr decomposition under different environmental conditions (within/above surrounding litter layer) for 48 temperate tree species and related them to an important leaf functional trait, i.e. leaf toughness. Leaf toughness could predict the changes well in terms of amounts, but poorly in terms of concentrations. Changes of 7 out of 10 litter chemical traits during decomposition showed a significant phylogenetic signal notably when litter was exposed above surrounding litter. These phylogenetic signals in element dynamics were stronger than those of initial elementary composition. Overall, relatively hard-to-measure ecosystem processes like element dynamics during decomposition could be partly predicted simply from phylogenies and leaf toughness measures. We suggest that the strong phylogenetic signals in chemical ecosystem functioning of species may reflect the concerted control by multiple moderately conserved traits, notably if interacting biota suffer microclimatic stress and spatial isolation from ambient litter.

## Introduction

Leaf litter decomposition is an important ecosystem process [1–3]. Many studies focus on overall mass loss of leaf litter, but during the decomposition processes also chemical traits might change significantly due to the release of various chemical elements from or their immobilization in litter [2–5]. For instance, amounts of phosphorus (P), and sometimes sulphur (S) may increase during the initial stages of decomposition (possibly owing to import by microbes), followed by a decrease [6,7], while Ca, K, Mg and Mn amounts usually continuously decrease [1]. Concentration of N, P and Ca increase in most litter, while Mg and S remain constant and K sometimes decreases [1,6–9], see also [4,10,11]. Previous studies mainly focused on litter chemical transformation of single species [11–16] or of litter mixtures [12,17]. Many of these studies found differences among elements or ecosystems concerned [4,10,12,17], but see [1,8,12,18,19,20]. However, studies across many species comparing species traits to chemical litter transformation are still missing.

Ecosystem processes may be driven by plant traits [21]. Mass loss during decomposition, for instance, can often be represented by easy-to-assess ‘soft’ leaf trait values [22]. However, few studies have tried to examine whether and how the patterns of change of litter chemical traits during decomposition across species can be predicted from species traits. Changes of litter chemical traits during decomposition are considered to be determined by the initial litter traits and the nutrient availability to decomposers [3,6,23,24]. Moreover, litter chemical changes might also link to initial litter physical traits, such as litter toughness (usually termed as leaf tensile strength). This physical soft trait was proven to have strong negative effects on the decomposition rates across multiple species [25,26]. In fact, given that leaf and litter toughness are strongly correlated, leaf toughness was usually chosen as an indicator of physical after-life litter quality and related to litter decomposition rate [10,25,26]. However, no study has systematically tested whether litter or leave toughness is a good predictor for the changes of multiple litter chemical traits during decomposition. Here, we hypothesized that leaf toughness might be a good driver for the changes of litter chemical traits during decomposition.

Functional traits may be more similar among close than among distantly related species (phylogenetic signal, e.g. [27]) and it has been suggested that phylogenetic structure of communities may influence their ecosystem functioning (e.g. [28]). The phylogenetic signal in species functional traits may hence translate into a phylogenetic signal in the ecosystem functioning of species, including in the chemical transformation of plant litter. Phylogenetic signal of species functioning might be weaker than that of traits as functioning is related to phylogeny only indirectly via the phylogenetic signal in traits [28]. However, functioning may also show a comparatively stronger phylogenetic signal if functioning results from multiple traits each shows a moderate signal. To our knowledge there exist few of any assessments of phylogenetic signals in ecosystem functioning of species [29] and no direct comparisons with functional traits. For instance, we do not know whether elementary changes during decomposition show stronger or weaker phylogenetic signal than elementary concentrations of the fresh litter. We here hypothesize that phylogenetic signals are weaker in traits (elementary concentrations) than in processes (elementary changes during decomposition).

Effect of traits or phylogenetic lineages on ecosystem function might be weakened or strengthened in particular environments. Strong abiotic stress has been suggested to filter out trait states within lineages [30] and these are putatively the ancestral trait states. Ancestral traits may correspond to ancestral functioning [31]. More generally, species traits may be particularly important for ecosystem functioning under abiotic stress [32]. For decomposition processes such an effect of abiotic stress is particularly plausible given the effect of stress decomposers [14,23], decomposition [12,33–37] and specifically on elementary dynamics [12,13]. However, to our knowledge the effect of environments on the strength of phylogenetic signal in ecosystem functioning has not been studies so far. For instance, we do not know whether elementary changes during decomposition show stronger or weaker phylogenetic signal when exposing the litter to more stressful environments. This would require comparative studies of elementary changes during decomposition for many plant species, which to our knowledge do not exist. We hypothesize that elementary changes during decomposition depend on microenvironment and that phylogenetic signal is stronger when micro-environmental stress is high.

To test our hypothesis we conducted a 1-yr litter bag experiment involving 48 temperate tree species litter under two contrasting environments: being decomposed “aboveground” versus “belowground”. For the aboveground treatment, litter bags were suspended in the air [38] and the decomposition process was presumably mainly affected by abiotic factors (see above) but not directly by soil microbe or invertebrates [38]. For the belowground treatment, litter bags were put on the soil surface and covered by a layer of a soil-litter mixture and abiotic stress was low and decomposition processes were mainly influenced by biotic factors, i.e. the microbial decomposition. We measured the leaf toughness and litter chemical traits before and after 1-yr decomposition and quantified the changes of litter chemical traits during decomposition in terms of both the amounts and the concentrations. Then we related the leaf toughness to the litter chemical traits and their changes, and examined the phylogenetic signals for leaf toughness, initial litter chemical traits and their changes during decomposition. We accounted for elementary changes in terms of both amounts and concentrations of different elements.

## Materials and Methods

### Ethics statement

Since all species are located on Beijing Botanical Garden (BJBG), the Chinese Academy of Sciences, permission from director of BJBG to enter the garden and collect plant material was provided before conducting this research.

### Study site and species selection

The study was conducted in the Beijing Botanical Garden (BJBG), China (116.216°E, 39.992°N, a.l.s. 76 m), with a history of more than 80 years. This site has a rather dry, monsoon-influenced humid continental climate, characterized by hot, humid summers and cold, windy, dry winters. The mean annual temperature is 11.8°C and the mean annual precipitation is 638.8 mm. The selected species in our study were all woody plants because woody plants produce much larger amounts of litters every year than other growth forms. Among the woody species, Rosales constituted a particularly important clade both in species numbers and in natural abundance in this temperate area. Therefore, we selected 23 angiosperm species within the clades of Rosales. We also selected another 25 woody species outside the Rosales clade including one gymnosperm species (*Ginkgo biloba*) based on their representation in the study region and the availability of litters in BJBG (full species list can be seen in S1 File). These species in total encompassed a wide range of temperate tree species and formed a good dataset to examine the phylogenetic similarities of litter chemical trait changes during decomposition [39].

### Leaf litter decomposition experiment

Leaf litter was sampled by either gently shaking the branches of at least five individuals of each species or from the ground below them in order to obtain newly senesced (i.e. still undecomposed) leaves. Litters were air-dried for at least two weeks at room temperature and five sub-samples for each species litter were selected for initial trait measurements and initial water content of the air-dry litter (for the estimation of initial dry weight). After initial trait measurements, each species litter was placed into 10 nylon litter bags respectively: 5 replicates for each treatment (see below). The sizes of litterbags were 10 × 15 cm, 15 × 20 cm, 15 × 25 cm, depending on the leaf size of different species. The mesh size was 1 mm for all the litterbags. Each litter bag was filled with 2 ± 0.1 g pre-weighed litters and sealed with staples.

Contrasting environments were achieved by either suspending the litter bags in the air (aboveground treatment) or putting litterbags on the ground covered by soil-litter mixtures (belowground treatment). The aboveground and belowground treatments were significantly different in the following aspects. Specifically, litters in the aboveground treatment were exposed mostly to by abiotic processes, such as leaching, physical fragmentation, thermal decomposition and/or photodegradation (see Introduction). On the other hand, litters in belowground treatment were directly exposed to soil microbes and soil invertebrates which exist in the soil-litter mixtures, but were not exposed to solar radiation (shaded by soil-litter mixtures). Therefore, mass loss and litter chemical changes in belowground treatment are expected to be mainly determined by biotic processes, i.e. microbial decomposition. In addition, there might also be differences in temperature and moisture between the aboveground and belowground litter bags.

The litter bed was built in a quiet and open space in BJBG. We cleared the aboveground vegetation and ploughed the soil surface (0–5 cm) of the whole litter bed (3 × 10 m) and evenly mixed the soil with an additional litter mixture collected from several areas of BJBG. This litter bed was enclosed by a wooden board covered by several metal meshes, which was used to suspend the litter bags aboveground. The metal mesh was about 25 cm above the litter layers in the litter bed and the size of the metal mesh was 5 cm. Litter bags were randomly arranged on the ground or on the metal meshes. For the aboveground treatment, we fixed the litterbags tightly onto the metal mesh in order to decrease the physical fragmentation by wind. The whole experiment ran for 1 year, because temperate tree species will produce huge amounts of fresh litters every year and the newly fallen litters may alter the chemical changes of litter from the previous year. We only harvested our litter bags once after 1 year. The harvested litter in each litter bag was carefully picked out and contaminants such as soil, little stones, grass roots and visible invertebrates were removed. Then we used a 2 cm sieve to separate the remaining litters into big and small pieces. These two parts were put in the paper bags separately for later dry mass measurements. Those two parts of decomposed litters were then oven-dried at 75°C for 48 hr and weighed. We documented the two weights and used the proportion of bigger particles to describe the fragmentation of the remaining litter i.e. the fragmentation index, and the initial fragmentation index for all species litters approximately equaled 1. The sum of those two weights was used to calculate the percentage mass loss of each litter bags.

### Leaf toughness and litter chemical trait measurements

One important functional trait, i.e. leaf tensile strength, was measured from green leaves of the respective species. We always collected fresh, mature, non-senescent sun leaves without significant herbivory symptoms to measure leaf tensile strength for all species [40]. The leaf tensile strength, termed as leaf toughness in our study, was measured as the force needed to break the leaf, expressed by per unit of width of a leaf sample rather than per cross-sectional area, thus incorporating leaf thickness as a component of tensile strength (unit: N cm^-1^). The measurements were taken following [41]. Moreover, litter chemical traits were measured on the initial litters and decomposed litters respectively. These included C, N, P, S, K, Ca, Mg, Mn, Fe and Zn. The C and N concentrations were determined by oven drying the litter at 75°C overnight with subsequent grinding using a modified ball mill [42]. The ground plant materials were analyzed on an automated elemental analyzer. The other element concentrations were analyzed by inductively coupled plasma emission spectroscopy (Perkin Elmer Optima 3000 ICP Spectrometer, Waltham, MA).

### Statistical analysis

We characterized the litter chemical changes during decomposition by two different variables: the change in the amounts and the change in the concentrations, both of which are considered to be important from an ecological point of view [12]. Then, we calculated the changes in the amounts by multiplying the concentration of a particular element after 1 yr decomposition by the remaining dry mass, which was expressed as a percentage of the initial amount of that particular element in the initial litters [17,43]. For each particular element, the change in the amount, which was calibrated by the remaining dry mass, represented the remaining amount of that particular element in the remaining litters after 1 year decomposition divided by the initial amount. Thus, values < 1 indicated a decrease and values > 1 an increase in the absolute amounts. Moreover, larges values in the changes of the amounts indicate that that particular element is released more slowly or (if > 1) even accumulated after 1 year of decomposition. We calculated the change in the concentrations by dividing the concentration of a particular element after 1 yr decomposition by the initial concentration. The change in the concentrations was the percentage of the initial concentration. The change in the concentration was calculated as for the change in the amount, but without calibration for remaining dry mass. Data were tested using a Shapiro-Wilk’s *W*-test to examine the assumptions of normality before the analyses, and non-normal data were log transformed when needed. One-way ANOVA was used to examine the difference in mass loss, fragmentation, litter chemical changes during decomposition between aboveground and belowground treatments. Moreover, a linear regression model was used to test whether leaf toughness was a good predictor for the litter chemical changes under both treatments. We verified the residuals for all the regression analyses, removed the extreme values (outliers) from the dataset and did the regression analyses again. Analyses were conducted in R (version 3.2.0) and in Statistica 7.0.

In addition, we estimated a plant phylogeny including 48 temperate tree species using ‘Phylomatic’ software online (http://phylodiversity.net/phylomatic/html/pm2_form.html, see also [39]. The species names and the taxonomic levels followed Angiosperm Phylogeny Group III [44]. For resolving polytomies, randomization was carried out with the help of the function ‘multi2di’ in the package ‘picante’ [45,46]. Branch length in the Phylomatic phylogeny was estimated using the ‘Bladj’ function in the ‘Phylocom’ software. In the end, we estimated the phylogenetic signals of the initial leaf toughness, initial litter chemical traits and the changes of litter chemical traits during decomposition under both aboveground and belowground treatments. We selected Blomberg’s *K* as a metric of phylogenetic signal [47], which was estimated using the function multiPhylosignal (Package picante) for all the chemical elements.

## Results

### Litter mass loss and litter fragmentation under different environmental conditions

**Fig 1:**
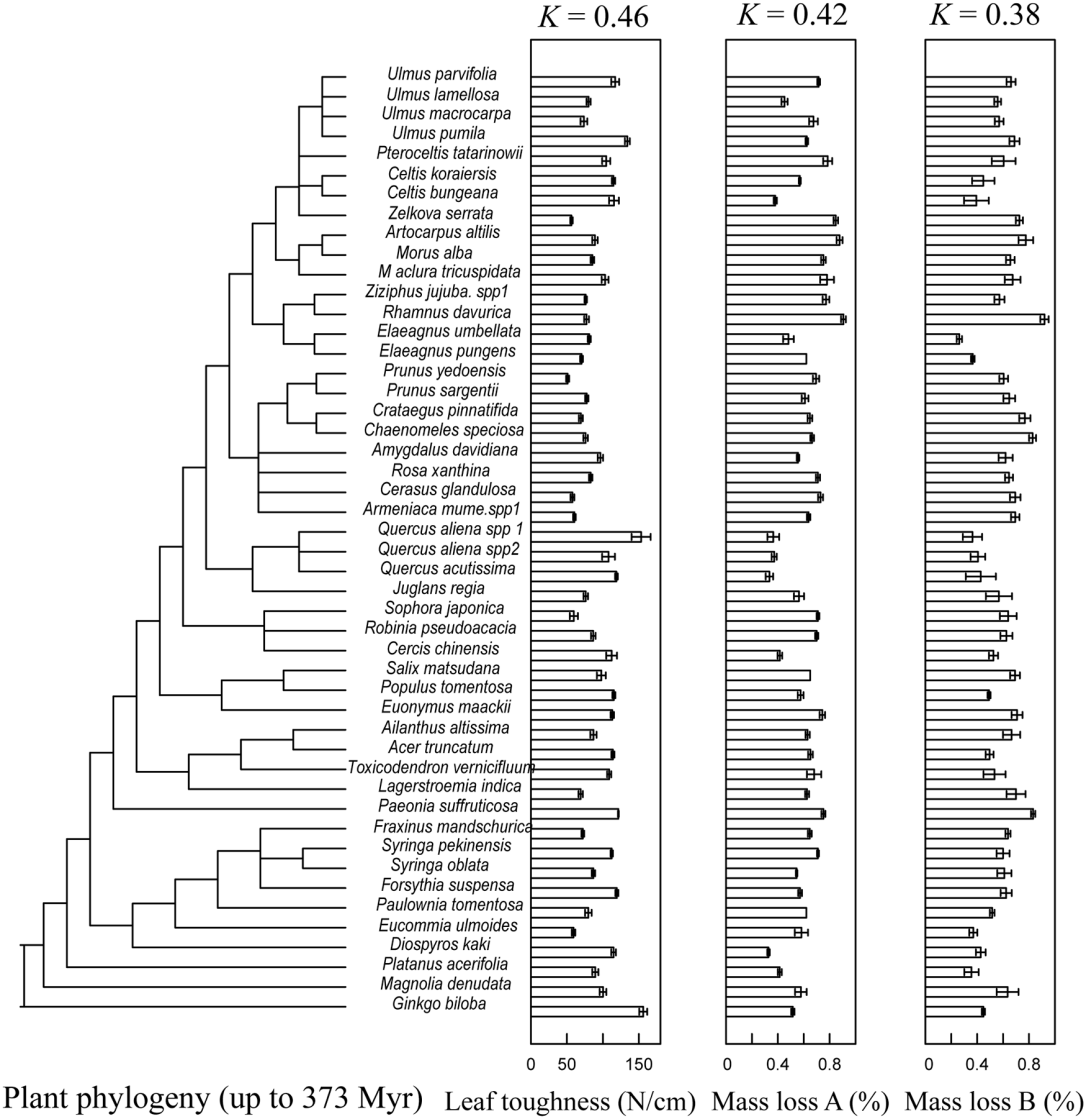
Leaf toughness, litter mass losses in aboveground and belowground treatments across our plant phylogeny of 48 species. Plant phylogeny came from the online software phylomatic and branch length was estimated using ‘Phylocom’ software. K represents the Blomberg’s K which is widely used to examine phylogenetic signals of traits and permits comparison among studies. The significance for the phylogenetic signals of leaf toughness and litter mass losses in aboveground and belowground treatments were 0.06, 0.05 and 0.05 respectively.

After 1 year of decomposition, litter mass losses in aboveground and belowground treatments were on average 0.620 and 0.589 respectively (Table 1), and litter fragmentation in aboveground and belowground treatments were on average 0.651 and 0.629, respectively. There was no significant difference in either litter mass losses or litter fragmentation between both treatments (One-way ANOVA, Table 1). Note that litter mass losses across species can be seen in Fig 1.

**Table 1:** Changes in litter mass, litter fragmentation index and, litter chemical traits during decomposition in aboveground and belowground treatments. Elements in the table are calculated as the fraction of the initial amount or concentration of particular element. For the change in elements (as fractions of final / initial), values < 1 indicate a decrease and values > 1 an increase in the absolute amounts (see main text). One-way ANOVA was used to examine the significance of differences between treatments.

Character changes during decomposition	Aboveground treatment (Mean ± SD)	Belowground treatment (Mean ± SD)	N	*F*	*P*
Change in litter mass (i.e. mass loss)	0.620 ± 0.139	0.589 ± 0.140	48	1.183	0.28
Change in litter fragmentation index	0.651 ± 0.151	0.629 ± 0.151	48	0.515	0.47
Change in amounts					
C	0.372 ± 0.133	0.365 ± 0.133	48	0.058	0.81
N	0.673 ± 0.247	0.754 ± 0.261	48	2.460	0.12
**P**	**0.363 ± 0.151**	**0.582 ± 0.211**	**48**	**34.39**	**< 0.01**
S	0.647 ± 0.348	0.643 ± 0.279	48	0.004	0.95
**K**	**0.097 ± 0.090**	**0.168 ± 0.153**	**48**	**7.641**	**< 0.01**
**Ca**	**0.492 ± 0.273**	**0.677 ± 0.228**	**48**	**12.93**	**< 0.01**
**Mg**	**0.161 ± 0.107**	**0.484 ± 0.229**	**48**	**78.29**	**< 0.01**
**Mn**	**0.758 ± 0.460**	**1.279 ± 0.648**	**48**	**20.59**	**< 0.01**
Fe	2.709 ± 1.337	2.474 ± 1.157	48	0.845	0.36
**Zn**	**4.228 ± 2.956**	**1.311 ± 0.788**	**48**	**43.64**	**< 0.01**
Change in concentrations					
**C**	**0.982 ± 0.051**	**0.883 ± 0.045**	**48**	**100.8**	**< 0.01**
N	1.801 ± 0.370	1.855 ± 0.346	48	0.554	0.46
**P**	**0.963 ± 0.229**	**1.445 ± 0.341**	**48**	**66.09**	**< 0.01**
S	1.753 ± 0.727	1.620 ± 0.565	48	1.000	0.32
**K**	**0.239 ± 0.182**	**0.378 ± 0.285**	**48**	**9.167**	**< 0.01**
**Ca**	**1.263 ± 0.458**	**1.680 ± 0.247**	**48**	**29.34**	**< 0.01**
**Mg**	**0.413 ± 0.164**	**1.169 ± 0.330**	**48**	**202.7**	**< 0.01**
**Mn**	**2.212 ± 1.256**	**3.404 ± 1.962**	**48**	**12.57**	**< 0.01**
**Fe**	**7.195 ± 2.585**	**6.133 ± 2.361**	**48**	**4.418**	**0.04**
**Zn**	**11.48 ± 5.720**	**3.253 ± 1.562**	**48**	**92.46**	**< 0.01**

### Changes of litter chemical traits during decomposition under different environmental conditions

The amounts of C, N, P, S, K, Ca and Mg decreased, but Fe and Zn increased after decomposition in both aboveground and belowground treatments (Table 1). The change in the amounts of P, K, Ca, Mg, Mn and Zn differed significantly between aboveground and belowground treatments (One-way ANOVA, Table 1). However, no significant difference was detected in C, N, S and Fe (Table 1). The concentrations of N, S, Ca, Fe, Mn and Zn increased, while the concentration of C, K and Mg decreased (Table 1). The change in the concentration of most chemical elements differed significantly between aboveground and belowground treatments except for N and S (One-way ANOVA, Table 1).

### Relationships between leaf toughness and changes of litter chemical traits during decomposition in aboveground and belowground treatments

**Fig 2:**
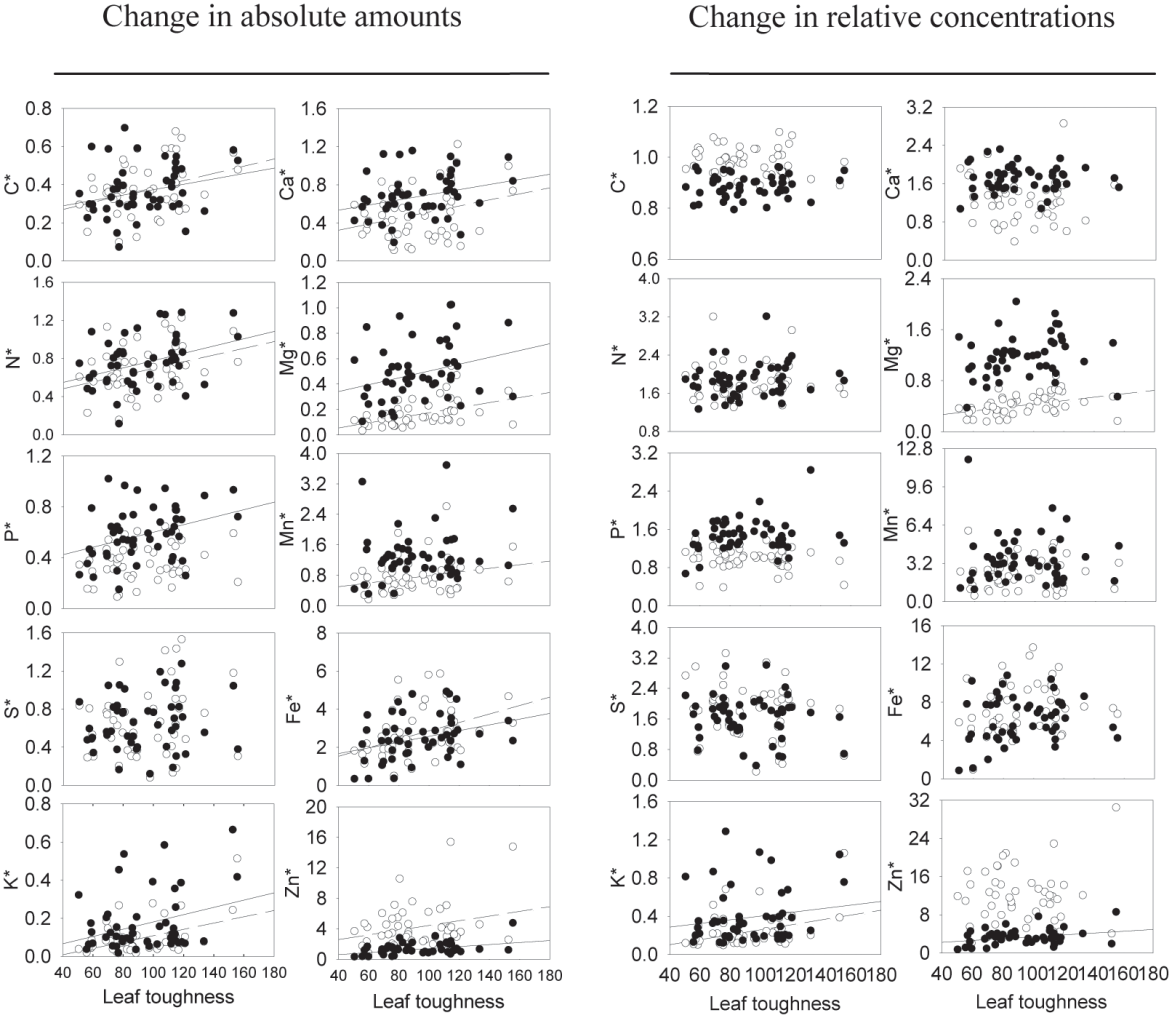
Relationships between leaf toughness and litter chemical trait changes (as fractions of final / initial) in amounts or in concentrations under different environment conditions. * stands for the changes of litter chemical traits, including both the amount and the concentration changes. Values < 1 indicate a decrease and values > 1 an increase in the absolute amounts (see main text). Each point represents a single species in our study. Circle points and dashed lines correspond to the aboveground treatment; and solid points and solid lines correspond to the belowground treatments. Significant (P<0.05) and marginally significant (P < 0.10) correlations are shown as regression lines.

In terms of the changes in the amounts of chemical traits, there were positive correlations between leaf toughness and most litter chemical traits except for litter [P] and [S] in the aboveground treatment and litter [S] and [Mn] in the belowground treatment (Fig 2). However, in terms of the change in the concentrations of chemical traits, significant correlations between leaf toughness and changes of chemical traits during decomposition were only found in litter [K] in both treatments, in litter [Mg] in aboveground treatment and in litter [Zn] in belowground treatment (Fig 2).

### Phylogenetic signals of initial leaf toughness, initial litter chemical traits and changes of litter chemical traits during decomposition under different environmental conditions

There was a marginally significant phylogenetic signal in the initial leaf toughness (Fig 1: K = 0.46, *P* = 0.06), and a significant phylogenetic signal was also found in 5 out of 10 initial chemical traits, i.e. for [C], [N], [S], [K] and [Ca] (Table 2, P < 0.05); but not for [P], [Mg], [Mn], [Fe] and [Zn] (Table 1, P ≥ 0.19). Moreover, after 1 year decomposition, there was a significant phylogenetic signal in the changes of [C], [S] and [K] in both aboveground and belowground treatments, but no significant phylogentic signal was found in the changes of [N], [P] and [Fe] in either above- or belowground treatment (Table 2). In addition, for the amount changes, there was also a significant phylogenetic signal in the changes of [Mg] and [Zn] in the aboveground treatment; for the concentration changes, a significant phylogenetic signal was also found in the changes of [Mg] and [Mn] in the aboveground treatment (Table 2).

**Table 2:** Tests for phylogenetic signals on initial values (as concentrations in mg/g) and the changes of litter chemical traits in absolute amounts and concentrations respectively, after 1-yr decomposition. K represents the Blomberg’s K which is widely used to examine phylogenetic signals of traits and permits comparison among studies. *P* represents the statistical significance of phylogenetic signal following the approach of [47]. *P* < 0.05 reflects close relatives being more similar than expected by chance [48].

Litter traits	Initial value		The amount changes				The concentration changes			
			Aboveground		Belowground		Aboveground		Belowground	
	*K*	*P*	*K*	*P*	*K*	*P*	*K*	*P*	*K*	*P*
**C**	**0.52**	**<0.01**	**0.41**	**0.05**	**0.40**	**0.05**	**0.44**	**<0.01**	**0.44**	**0.05**
**N**	**0.42**	**0.01**	*0*.*37*	*0*.*07*	*0*.*36*	*0*.*08*	0.26	0.49	0.23	0.70
**P**	0.34	0.30	*0*.*33*	*0*.*08*	*0*.*39*	*0*.*09*	0.31	0.38	0.19	0.90
**S**	**0.81**	**0.02**	**0.51**	**<0.01**	**0.44**	**0.02**	**0.63**	**0.01**	**0.68**	**<0.01**
**K**	**0.50**	**0.05**	**1.98**	**<0.01**	**0.49**	**0.02**	**1.72**	**<0.01**	**0.54**	**0.01**
**Ca**	**0.34**	**0.05**	**0.39**	**0.02**	*0*.*36*	*0*.*09*	0.36	0.06	0.24	0.72
**Mg**	0.26	0.53	0.33	0.15	0.27	0.37	**0.37**	**0.05**	0.28	0.73
**Mn**	0.41	0.19	*0*.*49*	*0*.*09*	0.37	0.16	**0.42**	**0.02**	0.27	0.45
**Fe**	0.33	0.43	0.41	0.13	0.29	0.26	0.36	0.09	0.29	0.27
**Zn**	0.35	0.33	**1.03**	**0.02**	0.54	0.31	0.58	0.09	0.32	0.69

## Discussion

### Changes of litter chemical traits during decomposition across species

The amounts of most litter chemical traits (C, N, P, S, K, Ca and Mg) decreased after 1 year of decomposition, indicating a net release of those elements from leaf litters to the temperate ecosystems. However, the other three litter chemical traits (Mn, Fe and Zn) showed a net accumulation after 1 year of decomposition. These results indicate that leaf litters of temperate tree species are still the source of most nutrient elements, but a sink for several metal elements. Our results on the changes of litter chemical traits during decomposition across 48 temperate tree species are in agreement with studies on the nutrient dynamics of single species, such as the silver birch, Norway spruce or multiple tropical forest species [15,16,24,49,50], but only in part consistent with the study in the Karri forest of south-western Australia [1], which showed that leaf litters of 3 out of 5 species accumulated phosphorus after 1 year of decomposition. However we did not find any evidence of phosphorus accumulation after 1 year of decomposition. The concentrations of most litter chemical traits (N, P, S, Ca, Mn, Fe and Zn) increased relative to the initial concentrations, but the concentrations of C and K decreased after 1 year of decomposition. These chemical trait changes across species were in agreement with studies on native or invasive species litters in Hawai’i [51], but only in part consistent with studies in the Karri forest of south-western Australia [1], which showed relatively constant concentrations of Mg and S. In contrast, we found significant increases in [Mg] and [S] after 1 year of decomposition. Possibly, the relatively short duration of decomposition in our study may explain the observed increase in [Mg] and [S].

### Leaf toughness as a predictor for the changes of litter chemical traits during decomposition

Our results showed that leaf toughness was a good predictor for the changes of litter chemical traits in terms of amounts. In species with tougher leaves, the amounts of elements after decomposition were higher in the remaining litters. Leaf toughness can enhance the litter resistance to soil decomposers [10,25,40], retard the leaf litter mass losses and hence lead to slower losses in the amounts of elements during decomposition. Therefore, there was a consistent correlation between leaf toughness and the change in the amounts of litter chemical traits across species. These results indicate that leaf toughness determines the amounts of litter nutrient release from litter back to the environment. They also provide us a physical trait that is easily and inexpensively measurable to predict the amounts of litter chemical changes during decomposition, the latter being relatively difficult and expensive to measure. However, leaf toughness was a poor predictor for the changes in litter chemical traits in terms of the concentration changes. One explanation might be that litter chemical trait changes in terms of the concentrations are not only determined by initial litter quality and the decomposers, but also determined by the relationships among the changes of multiple chemical traits during decomposition. For example, a large decrease of the amounts of carbon might increase the concentrations of other chemical traits, even though in fact the latter also decrease in absolute amounts during decomposition.

### Phylogenetic signal as a predictor for the similarities of the changes of litter chemical traits during decomposition among species

Our results showed that there were significant phylogenetic signals of initial litter C, S and K, and the changes of those three litter chemical traits also showed significant phylogenetic signal, regardless of the way of quantifying litter chemical trait changes or the environmental conditions for litter incubation. For C, there was a large decrease in the amount during decomposition, while the concentrations of C also decreased only slightly. Together these changes led to the convergence of litter C across species and the significant phylogenetic signals in initial litter C remained but became weaker during decomposition [18]. This matches the fact that chemical traits will become more and more similar across species with the progression of litter decomposition. However, the detected strong phylogenetic signal of S and K during decomposition became even stronger than the initial phylogenetic signals. Moreover, our results also showed that there was a significant phylogenetic signal in initial litter N, but there was no phylogenetic signal in the changes of litter N during decomposition. This might be due to the relatively small decrease of initial amounts of N and the increase of the concentration of nitrogen during decomposition, leading to the disappearance of phylogenetic signal of litter N during decomposition. In addition, there was neither significant phylogenetic signal in the initial litter P and Fe, nor in the changes in P and Fe. These results indicate that there was no similarity among closely related species in P and Fe or their changes during decomposition.

Overall, phylogenetic signals in elementary dynamics seem to be stronger than those in initial concentrations of the same elements (in particular above ground). Such a situation is surprising at first sight as ecosystem functioning of species such as elementary dynamics during decomposition is related to phylogenetic position of species only indirectly, via differences in functional traits. Such an indirect relationship should be weaker than the more direct one between phylogenetic position and trait states. However, we can imagine a scenario that does produces stronger phylogenetic signals in ecosystem processes than in traits. Imagine a process that depends on the *combined* effect of multiple traits, each of which with a moderate phylogenetic signal. A major change in this process hence requires a *concerted* change of all traits together. Given their phylogenetic signals, such a concerted major change is likely to happen only among distant relatives belonging to different lineages, even if each of the traits may show some minor variation within each of the lineages. The phylogenetic signal at the level of the ecosystem process might hence be stronger than that at level of the individual traits involved. In the present case, a major change among species in the dynamics of a given element may require a concerted, major change of both the composition of this element, of other elements and of leaf toughness and may hence be unlikely to happen among closely related species.

### Role of environmental conditions in predicting the changes of litter chemical traits during decomposition

Environment significantly influenced the litter chemical changes in terms of amounts during decomposition across species (Table 1). Our results showed that the changes in the amounts of most litter chemical traits, except for C, N, S and Fe, differed significantly between the aboveground and belowground treatments and in all cases the amounts of litter elements in the aboveground treatment decreased or increased faster than those in belowground treatments. About 80–90% of the initial amounts of K was released after 1 yr decomposition, and a similar phenomenon was observed in a broad range of forest ecosystems [52].This might be due to fast-turnover elements such as K remain in plant residues as water soluble salts [50] and the changes in K might be dominated by the leaching process in ecosystems [52]. Together our results suggest that abiotic processes might contribute more to the litter chemical changes during decomposition than microbial activity. However, the small mesh size of the litter bags in our study might have excluded certain groups of invertebrates that might also be important for leaf litter decomposition processes, especially fragmentation. Therefore, the biotic contribution to litter chemical changes during decomposition might have been underestimated in our study.

Environment also significantly influenced the litter chemical changes in the concentrations during decomposition across species (Table 1). Our results showed that the increases in P, Ca, Mg and Mn were bigger in the belowground treatment than that in the aboveground treatment. The increase in concentrations of most chemical elements was suggested to be due to the immobilization of the amounts in litter by microbial biomass and humic substances [12]. Larger increases in the concentrations in belowground treatments may be due to the stronger immobilization by soil microbes; however in the aboveground treatment they can also be due to the same immobilization by microbes (possibly airborne microbes colonizing the litter) in combination with stronger leaching. The latter was also confirmed by the decrease of Mg concentration in the aboveground treatment, in contrast to an increase of Mg concentration in the belowground treatment. Indeed the decrease of Mg concentration has been considered to be determined by leaching rather than biological processes [53]. Moreover, our results also showed that the increase in Fe and Zn concentrations in the belowground treatment was even smaller than that in the aboveground treatment, which was perhaps due to the exclusion of soil invertebrates (see above). The mechanisms for the accumulation of Fe or Zn were suggested to be abiotic absorption on humified litters or an effect of accumulation of metal elements in fungi [54–56]. Our results indicate that the concentrations of Fe and Zn might also increase via other processes, such as the input with through fall, biological translocation by fungi from deeper soil layers [9] or possibly by microbes from the air. Different fungi, i.e. airborne fungi versus soil fungi in aboveground and belowground treatments respectively, might determine how litter chemical trait changed during decomposition [57].

We found that the phylogenetic signal of elementary dynamics tends to be stronger above ground than below ground. Signals of plant phylogeny on decomposition of plant litter may result from litter of different plant lineages differentially sorting decomposers. The sorting of decomposers by a type of litter may be more powerful if decomposers are at their physiological limits, i.e. under abiotically more stressful conditions. Filtering by a type of litter may also be more powerful the litter is spatially isolated from other types of litter, as decomposers cannot easily emigrate to use adjacent complementary litter types nor spill over from such adjacent litter types. Indeed, above-ground treatments were abiotically more extreme and spatially more isolated from the surrounding litter than below ground treatments. Together this might render sorting of decomposers by plant traits more powerful. These traits are to some degree phylogenetically conserved, resulting potentially in comparatively strong phylogenetic signals in decomposer biota, and in the elementary dynamics induced by these decomposers. We stress that this interpretation remains speculative and each of the mechanisms suggested needs to be tested in future research.

## Conclusion

Our study was the first to predict litter chemical changes during decomposition from other leaf traits and their similarities by their phylogenetic relatedness. These results thereby contribute to the growing literature on litter chemical changes during decomposition, which has received much less attention than the mass loss, or *k*-values in former decomposition studies. Our results indicate that the dynamics of key ecologically relevant elements during decomposition in terms of the amounts can be well predicted using leaf toughness, while changes in these elements in terms of their concentration were poorly predicted by leaf toughness. These results might improve our confidence to predict different aspects of element cycling in ecosystems using plant functional traits in future. Moreover, litter chemical trait changes showed interesting evolutionary patterns and we found significant phylogenetic signals in 7 out of 10 chemical traits. These results provide us a possibility to predict the ecosystem functioning in an evolutionary perspective.

## Supporting Information

S1 File Species list. Nomenclature follows APG III [34].
(PDF)

S1 Table Initial chemical traits across 48 species. (PDF)

S2 Table Chemical traits after 1-yr decomposition under aboveground treatment. (PDF)

S3 Table Chemical traits after 1-yr decomposition under belowground treatment. (PDF)

S4 Table Litter mass loss, fragmentation index and leaf toughness across 48 species. (PDF)
