# Supplementary material for: Evolutionary Position and Leaf Toughness Control Chemical Transformation of Litter, and Drought Reinforces This Control: Evidence from a Common Garden Experiment across 48 Species
Source: PLoS One. 2015 Nov 17;10(11):e0143140. doi: 10.1371/journal.pone.0143140 (PMC4648592; doi:10.1371/journal.pone.0143140)
Supplement: S1 File — Nomenclature follows APG III [34]. (PDF) [file pone.0143140.s001.pdf]

S1File. Species list

Nomenclature follows APG III (The Angiosperm Phylogeny Group, 2009).

Gymnosperm

Ginkgoales

Ginkgoaceae

Ginkgo

1.*Ginkgo biloba* Linnaeus

Pinales

Pinaceae

Pinus

2.*Pinus bungeana* Zucc. et Endi.

3.*Pinus armandii* Franch.

4. *Pinus tabulaeformis* Carr. var. *mukdensis* Uyeki

Angiosperm

Magnoliids

Magnoliales

Magnoliaceae

Magonlia Linn

5. *Magnolia denudata* Desr.

Eudicots

Proteales

Platanaceae

Platanus

6. *Platanus* × *acerifolia* (Ait.) Willd.

Core Eudicots

Saxifragales

Paeoniaceae

Paeonia

7. *Paeonia suffruticosa* Andr.

Rosids ----- fabids

Celastrales

Celastraceae

Euonymus

8. *Euonymus maackii Rupr*

Malpighiales

Salicaceae

Populus

9.*Populus tomentosa* Carr.

Salix

10.*Salix matsudana* var. *matsudana f. pendula* Schneid.

Fabales

Fabaceae

Styphnolobium

11.*Sophora japonica* Linn.

Cercis

12. *Cercis chinensis* Bunge

Robinia

13. *Robinia pseudoacacia* L.

Fagales

Fagaceae

Quercus

14. *Quercus aliena* var. *pekingensis*
15. *Quercus aliena* var. *acuteserrata*
16. *Quercus acutissima* Carr.

Juglandaceae

Juglans

17. *Juglans regia*

Rosales

Elaeagnaceae

Elaeagnus

18. *Elaeagnus umbellata* Thunb.
19. *Elaeagnus pungens* Thunb.

Moraceae

Maclura

20. *Maclura tricuspidata* Carrière

Morus

21. *Morus alba* Linn.

Artocarpus

22. *Artocarpus altilis* (Parkinson) Fosberg

Rhamnaceae

Rhamnus

23. *Rhamnus davurica* Pall.

Ziziphus

24. *Ziziphus jujuba* var. *spinosa* (Bunge) Hu

Rosaceae

Chaenomeles

25. *Chaenomeles speciosa* (Sweet) Nakai

Crataegus

26. *Crataegus pinnatifida* Bge.

Amygdalus

27. *Amygdalus davidiana* (Carrière) de Vos ex Henry

Rosa

28. *Rosa xanthina* Lindl.

Prunus

29. *Prunus sargentii* Rehder.
30. *P*runus yedoe*nsis* Matsum.

Armeniaca

31. *Armeniaca mume* var. *bungo*
Cerasus
32. *Cerasus glandulosa* (Thunb.) Lois.
Ulmaceae
Pteroceltis
33. *Pteroceltis tatarinowii* Maxim.
Ulmus
34. *Ulmus lamellosa* C. Wang et L. Chang
35. *Ulmus pumila* L.
36. *Ulmus macrocarpa* Hance
37.*Ulmus parvifolia* Jacq.
Celtis
38. *Celtis bungeana* Bl.
39. *Celtis koraiersis* Nakai
Zelkova
40. *Zelkova serrata* (Thunb.) Mak.
Rosids ---- malvids
Myrtales
Lythraceae
Lagerstroemia
41. *Lagerstroemia indica* L.
Sapindales
Aceraceae
Acer
42. *Acer truncatum* Bunge
Anacardiaceae
Toxicodendron
43. *Toxicodendron vernicifluum* (Stokes) F. A.Barkley
Simaroubaceae
Ailanthus
44. *Ailanthus altissima* (Mill.) Swingle
Asterids
Ericales
Ebenaceae
Diospyros
45. *Diospyros kaki* Thunb.
Asteroids --- lamiids
Lamiales
Oleaceae
Forsythia
46. Forsythia suspensa (Thunb.) Vahl
Syringa
47. *Syringa oblata* Lindl.
48. *Syringa pekinensis* Rupr.

Fraxinus

49. *Fraxinus mandschurica* Rupr.

Eucommiaceae

Eucommia

50. *Eucommia ulmoides* Oliver

Paulowniaceae

Paulownia

51. *Paulownia tomentosa* (Thunb.) Steud.

Reference:

The Angiosperm Phylogeny Group. An update of the angiosperm phylogeny group classification for the orders and families of flowering plants: APGIII. *Bot J Linn Soc.* **161**:105-121.
