# Supplementary material for: Evolutionary Position and Leaf Toughness Control Chemical Transformation of Litter, and Drought Reinforces This Control: Evidence from a Common Garden Experiment across 48 Species
Source: PLoS One. 2015 Nov 17;10(11):e0143140. doi: 10.1371/journal.pone.0143140 (PMC4648592; doi:10.1371/journal.pone.0143140)
Supplement: S1 Table — (PDF) [file pone.0143140.s002.pdf]

S1 Table. Initial chemical traits across 48 species

species	N（%）	C（%）	Ca（mg/g）	Fe（mg/g）	K（mg/g）	Mg（mg/g）	Mn（mg/g）	P（mg/g）	S（mg/g）	Zn（mg/g）
ginkgo_biloba	1.33	45.49	44.17	0.54	1.84	9.05	0.03	4.10	0.12	0.01
magnolia_denudata	0.91	39.94	42.64	0.54	2.92	5.75	0.04	1.44	0.03	0.02
platanus_acerifolia	1.21	46.58	19.45	0.48	5.66	3.12	0.11	1.38	0.09	0.02
diospyros_kaki	1.94	45.66	33.23	0.63	7.38	5.78	0.26	1.48	0.06	0.02
eucommia_ulmoides	1.18	46.25	19.92	0.64	11.33	3.90	0.08	1.31	0.07	0.04
tomentosa	1.66	45.11	31.73	0.66	8.61	4.97	0.05	1.94	0.05	0.03
forsythia_suspensa	0.87	48.71	16.79	0.40	6.89	2.96	0.15	2.58	0.03	0.02
fraxinus_mandschurica	1.44	44.79	30.23	0.94	6.25	4.03	0.05	1.13	0.06	0.03
syringa_oblata	0.94	46.24	16.76	0.49	7.59	3.45	0.03	2.72	0.04	0.02
syringa_pekinensis	1.23	45.50	29.52	0.56	5.81	5.08	0.05	1.10	0.05	0.02
paeonia_suffruticosa	0.97	42.96	34.17	0.68	3.86	4.33	0.03	1.64	0.03	0.02
lagerstroemia_indica	1.06	42.89	29.19	0.46	7.21	6.27	0.05	1.45	0.03	0.03
toxicodendron_vernicifluum	0.87	44.69	45.35	0.73	5.53	5.88	0.06	2.51	0.07	0.03
acer_truncatum	1.33	43.95	40.77	0.55	11.20	4.32	0.10	1.65	0.04	0.03
ailanthus_altissima	1.25	45.76	33.64	0.58	8.52	4.74	0.04	1.24	0.03	0.02
euonymus_maackii	1.25	42.79	49.91	0.50	4.76	6.28	0.05	6.91	0.16	0.03
populus_tomentosa	1.10	40.93	42.00	0.50	11.20	5.20	0.16	1.54	0.11	0.19
salix_matsudana	1.18	40.76	46.06	0.47	14.39	6.20	0.10	1.50	0.32	0.06
cercis_chinensis	1.35	46.27	23.98	0.44	8.51	3.20	0.02	1.81	0.03	0.02
sophora_japonica	2.23	44.71	37.72	0.94	9.10	4.11	0.08	2.15	0.06	0.03
robinia_pseudoacacia	2.05	43.62	43.88	0.49	4.71	5.40	0.05	1.78	0.05	0.03
juglans_regia	1.79	47.65	29.33	0.39	3.93	4.97	0.07	1.99	0.04	0.02
quercus_aliena var.pekingensis	0.77	45.39	25.92	0.63	3.88	3.41	0.64	1.59	0.02	0.04
quercus_aliena var.acuteserrata	0.78	46.40	26.97	0.43	3.80	3.87	0.32	1.08	0.02	0.02

quercus_acutissima	0.84	48.97	20.63	0.41	2.98	2.90	0.12	1.05	0.03	0.05
amygdalus_davidiana	1.22	45.12	53.19	4.35	8.71	8.49	0.23	2.45	0.06	0.12
rosa_xanthina	1.26	46.28	22.14	0.54	7.26	3.82	0.10	1.21	0.03	0.03
armeniaca_mume var.bungo	1.44	44.84	38.33	0.56	14.39	8.72	0.07	1.31	0.05	0.02
chaenomeles_speciosa	1.41	46.91	25.77	0.36	7.70	4.15	0.05	1.84	0.03	0.05
crataegus_pinnatifida	1.27	46.09	31.81	0.64	3.77	5.50	0.03	1.35	0.03	0.05
prunus_sargentii	0.86	43.75	46.17	0.67	2.01	10.48	0.13	1.43	0.03	0.02
prunus_yedoensis	0.78	44.29	42.98	0.48	1.72	9.86	0.07	1.10	0.02	0.02
cerasus_glandulosa	1.55	44.95	28.71	0.66	13.29	4.89	0.06	1.78	0.03	0.03
elaeagnus_pungens	2.00	49.16	20.09	1.37	4.24	3.79	0.18	1.11	0.04	0.13
elaeagnus_umbellata	1.61	48.70	19.03	0.60	7.18	4.46	0.18	1.01	0.04	0.01
rhamnus_davurica	1.93	42.48	37.50	1.31	16.39	3.57	0.09	1.65	0.04	0.03
ziziphus_jujuba var.spinosa	1.27	43.42	41.36	0.47	16.05	4.36	0.07	6.54	0.03	0.05
maclura_tricuspidata	1.43	40.85	52.75	0.50	9.17	5.08	0.06	1.87	0.03	0.04
morus_alba	1.64	39.50	40.60	0.66	17.28	6.62	0.08	2.86	0.06	0.04
artocarpus_altilis	1.02	39.46	45.79	1.50	24.61	5.51	0.04	2.64	0.04	0.03
pteroceltis_tatarinowii	1.38	37.08	35.73	0.56	18.07	17.25	0.04	1.69	0.04	0.05
zelkova_serrata	0.99	44.09	25.37	0.59	10.13	2.48	0.06	1.46	0.02	0.04
celtis_bungeana	0.95	43.38	47.95	0.58	5.47	1.95	0.08	1.08	0.03	0.05
celtis_koraiersis	0.65	37.65	70.49	0.73	6.89	5.30	0.06	1.41	0.02	0.02
ulmus_lamellosa	1.22	39.67	44.97	0.65	8.96	4.95	0.06	1.55	0.04	0.03
ulmus_pumila	1.17	39.53	42.30	0.70	12.66	4.57	0.06	1.48	0.04	0.03
ulmus_macrocarpa	1.17	42.43	29.12	0.25	8.00	3.99	0.02	1.75	0.04	0.02
ulmus_parvifolia	1.12	40.30	27.80	0.60	18.90	3.59	0.06	1.63	0.03	0.05
