# Supplementary material for: Evolutionary Position and Leaf Toughness Control Chemical Transformation of Litter, and Drought Reinforces This Control: Evidence from a Common Garden Experiment across 48 Species
Source: PLoS One. 2015 Nov 17;10(11):e0143140. doi: 10.1371/journal.pone.0143140 (PMC4648592; doi:10.1371/journal.pone.0143140)
Supplement: S2 Table — (PDF) [file pone.0143140.s003.pdf]

S2 Table. Chemical traits after 1-yr decomposition under aboveground treatment

species	N（%）	C（%）	Ca（mg/g）	Fe（mg/g）	K（mg/g）	Mg（mg/g）	Mn（mg/g）	P（mg/g）	S（mg/g）	Zn（mg/g）
ginkgo_biloba	2.07	44.50	66.47	3.61	1.93	1.38	0.08	1.70	0.07	0.26
magnolia_denudata	1.52	35.38	26.73	7.32	1.89	3.04	0.16	1.48	0.06	0.33
platanus_acerifolia	2.11	46.46	27.62	2.74	1.02	1.47	0.08	1.42	0.06	0.23
diospyros_kaki	2.64	45.87	46.71	4.17	1.24	1.60	0.11	1.31	0.06	0.36
eucommia_ulmoides	2.13	47.81	15.04	3.27	1.03	1.38	0.10	1.39	0.05	0.20
tomentosa	2.35	43.86	22.47	7.75	1.51	2.57	0.17	1.63	0.08	0.45
forsythia_suspensa	1.87	51.11	47.72	1.82	1.05	1.82	0.15	1.56	0.06	0.18
fraxinus_mandschurica	2.41	46.49	23.35	4.71	1.31	1.77	0.12	1.32	0.07	0.30
syringa_oblata	1.98	47.75	14.97	4.73	1.82	2.03	0.10	1.47	0.06	0.20
syringa_pekinensis	2.09	48.06	32.91	4.76	1.22	2.22	0.11	1.22	0.06	0.23
paeonia_suffruticosa	2.80	46.48	20.12	4.93	1.52	1.63	0.10	1.98	0.06	0.27
lagerstroemia_indica	2.30	40.09	64.21	3.10	1.51	1.94	0.12	1.79	0.06	0.25
toxicodendron_vernicifluum	1.86	44.24	43.35	5.08	1.20	2.00	0.17	2.40	0.07	0.44
acer_truncatum	2.11	41.41	65.16	3.48	1.34	1.34	0.12	1.36	0.06	0.32
ailanthus_altissima	2.17	46.15	12.39	2.71	1.11	1.23	0.08	1.58	0.05	0.22
euonymus_maackii	2.42	46.85	34.21	5.40	1.77	2.85	0.25	7.17	0.08	0.47
populus_tomentosa	1.46	38.49	60.60	4.54	1.60	2.24	0.15	1.25	0.04	0.35
salix_matsudana	1.92	41.95	61.16	6.04	1.64	2.31	0.18	1.47	0.06	0.42
cercis_chinensis	2.54	46.42	26.95	3.56	1.56	2.03	0.10	1.60	0.06	0.22
sophora_japonica	3.41	43.59	57.03	3.56	1.45	1.69	0.13	1.82	0.08	0.34
robinia_pseudoacacia	3.16	43.53	57.12	3.06	1.33	1.75	0.13	1.74	0.09	0.39
juglans_regia	2.78	44.57	50.13	3.50	1.24	1.51	0.11	1.59	0.07	0.26
quercus_aliena var.pekingensis	1.41	43.67	40.00	2.76	1.53	2.37	0.41	1.27	0.05	0.15
quercus_aliena var.acuteserrata	1.43	43.06	39.00	3.94	1.33	1.98	0.18	1.10	0.05	0.26

quercus_acutissima	1.42	43.37	32.09	2.98	1.12	1.55	0.12	0.97	0.05	0.18
amygdalus_davidiana	1.85	46.22	60.26	3.64	1.17	1.42	0.10	0.96	0.05	0.22
rosa_xanthina	2.72	46.86	40.11	2.18	1.06	1.08	0.10	1.66	0.08	0.50
armeniaca_mume var.bungo	1.86	44.25	72.33	4.56	1.56	1.48	0.13	1.12	0.06	0.34
chaenomeles_speciosa	2.63	48.29	29.69	3.32	1.21	1.23	0.10	1.90	0.07	0.26
crataegus_pinnatifida	2.39	45.51	39.79	3.84	1.35	1.36	0.09	1.54	0.08	0.29
prunus_sargentii	2.74	47.00	61.75	2.83	1.02	1.50	0.12	1.22	0.05	0.27
prunus_yedoensis	1.50	44.12	60.08	3.02	1.16	1.56	0.10	1.11	0.05	0.33
cerasus_glandulosa	3.04	42.71	54.52	3.80	1.45	1.71	0.13	1.96	0.07	0.34
elaeagnus_pungens	2.64	46.13	30.83	6.67	1.58	1.49	0.14	1.56	0.08	0.38
elaeagnus_umbellata	2.34	47.16	26.09	3.15	1.07	1.33	0.12	1.12	0.06	0.27
rhamnus_davurica	3.05	42.24	42.20	6.44	1.99	2.36	0.26	1.73	0.09	0.47
ziziphus_jujuba var.spinosa	2.88	45.97	25.26	3.65	1.36	1.42	0.11	2.40	0.08	0.68
maclura_tricuspidata	2.42	39.06	48.88	4.90	1.43	2.32	0.16	1.84	0.07	0.32
morus_alba	2.42	35.49	48.79	6.31	1.87	2.86	0.24	2.62	0.07	0.49
artocarpus_altilis	2.35	38.29	41.78	9.84	2.46	3.07	0.19	2.62	0.09	0.54
pteroceltis_tatarinowii	1.99	35.65	62.63	5.79	1.78	2.87	0.25	1.63	0.07	0.49
zelkova_serrata	1.64	41.28	34.78	3.68	1.60	2.14	0.11	1.64	0.06	0.27
celtis_bungeana	1.65	40.82	56.63	3.16	1.42	1.27	0.17	1.21	0.05	0.20
celtis_koraiersis	1.47	34.88	80.66	3.97	1.62	3.22	0.19	1.92	0.07	0.27
ulmus_lamellosa	2.09	36.11	36.34	4.80	1.72	2.23	0.14	1.70	0.07	0.30
ulmus_pumila	2.11	38.75	31.54	4.07	1.31	1.71	0.12	1.37	0.07	0.31
ulmus_macrocarpa	2.04	40.76	42.27	2.54	1.38	1.95	0.08	1.44	0.06	0.18
ulmus_parvifolia	1.98	36.53	33.44	6.89	1.91	2.26	0.17	1.61	0.09	0.56
