# Supplementary material for: Evolutionary Position and Leaf Toughness Control Chemical Transformation of Litter, and Drought Reinforces This Control: Evidence from a Common Garden Experiment across 48 Species
Source: PLoS One. 2015 Nov 17;10(11):e0143140. doi: 10.1371/journal.pone.0143140 (PMC4648592; doi:10.1371/journal.pone.0143140)
Supplement: S3 Table — (PDF) [file pone.0143140.s004.pdf]

S3 Table. Chemical traits after 1-yr decomposition under belowground treatment

species	N（%）	C（%）	Ca（mg/g）	Fe（mg/g）	K（mg/g）	Mg（mg/g）	Mn（mg/g）	P（mg/g）	S（mg/g）	Zn（mg/g）
ginkgo_biloba	2.45	42.97	66.36	2.25	1.37	4.82	0.11	5.31	0.08	0.07
magnolia_denudata	1.99	34.49	66.05	2.89	3.10	6.77	0.13	3.12	0.07	0.05
platanus_acerifolia	2.09	42.41	34.66	3.54	1.77	3.79	0.21	1.98	0.06	0.06
diospyros_kaki	2.64	40.88	63.15	2.50	1.42	4.33	0.36	1.99	0.07	0.06
eucommia_ulmoides	2.00	43.65	29.47	2.89	3.04	5.21	0.19	1.63	0.06	0.08
tomentosa	2.95	42.56	46.80	3.16	1.56	5.45	0.16	2.88	0.08	0.09
forsythia_suspensa	1.98	45.41	29.64	3.04	1.29	4.19	0.27	2.51	0.05	0.07
fraxinus_mandschurica	2.41	39.65	44.22	5.08	1.85	5.01	0.19	2.11	0.08	0.11
syringa_oblata	1.99	44.30	30.44	3.19	1.14	4.83	0.13	2.48	0.06	0.10
syringa_pekinensis	2.44	41.38	43.97	4.26	1.92	5.74	0.18	1.94	0.07	0.06
paeonia_suffruticosa	2.28	38.25	53.81	4.25	1.45	5.71	0.17	2.45	0.06	0.10
lagerstroemia_indica	1.94	38.41	65.68	3.52	2.28	5.21	0.18	2.06	0.05	0.09
toxicodendron_vernicifluum	1.40	39.94	54.28	3.85	2.03	5.98	0.17	3.12	0.06	0.08
acer_truncatum	2.13	38.31	59.98	5.15	3.16	5.93	0.23	2.08	0.06	0.10
ailanthus_altissima	2.39	39.62	56.16	4.83	2.93	5.61	0.22	1.98	0.06	0.09
euonymus_maackii	2.31	40.95	73.61	2.42	1.83	6.15	0.17	9.43	0.10	0.09
populus_tomentosa	2.12	37.47	66.19	3.46	1.72	4.69	0.30	2.11	0.06	0.31
salix_matsudana	2.37	36.72	83.79	3.58	2.22	5.23	0.31	2.61	0.12	0.17
cercis_chinensis	2.19	39.69	41.83	4.53	1.65	5.02	0.17	2.27	0.05	0.08
sophora_japonica	3.08	39.11	69.77	4.17	3.51	5.89	0.23	2.84	0.08	0.13
robinia_pseudoacacia	2.55	35.32	75.17	4.95	1.60	5.44	0.24	2.10	0.07	0.12
juglans_regia	2.96	45.14	44.03	1.63	1.35	4.55	0.16	2.36	0.06	0.08
quercus_aliena var.pekingensis	1.72	37.83	46.10	4.92	2.58	5.05	0.93	1.93	0.04	0.08
quercus_aliena var.acuteserrata	1.64	42.56	39.74	2.66	3.70	4.79	0.39	1.70	0.04	0.08

quercus_acutissima	1.67	44.36	35.00	2.15	3.09	3.99	0.20	1.53	0.04	0.08
amygdalus_davidiana	2.51	38.67	69.45	4.33	1.81	6.50	0.22	1.91	0.07	0.09
rosa_xanthina	2.43	44.32	46.17	2.20	1.38	3.69	0.17	1.81	0.05	0.10
armeniaca_mume var.bungo	2.25	35.46	73.81	6.03	3.66	8.50	0.27	1.96	0.06	0.10
chaenomeles_speciosa	2.71	42.17	48.00	2.62	1.48	4.84	0.19	2.84	0.07	0.10
crataegus_pinnatifida	2.25	37.82	57.56	4.90	2.19	5.40	0.19	2.26	0.06	0.08
prunus_sargentii	2.10	39.75	73.11	2.90	1.73	7.08	0.28	2.50	0.06	0.08
prunus_yedoensis	1.92	37.12	71.55	4.02	2.20	7.37	0.23	1.90	0.05	0.06
cerasus_glandulosa	2.91	39.54	30.22	0.49	10.68	7.19	0.06	1.16	0.06	0.02
elaeagnus_pungens	2.99	45.18	35.32	2.60	1.44	3.84	0.33	1.78	0.06	0.09
elaeagnus_umbellata	2.31	45.72	28.54	1.84	5.17	5.60	0.27	1.31	0.05	0.05
rhamnus_davurica	2.56	35.83	86.36	5.23	2.99	6.02	0.35	2.95	0.08	0.10
ziziphus_jujuba var.spinosa	2.49	37.89	64.67	4.26	1.76	5.38	0.21	9.74	0.06	0.15
maclura_tricuspidata	2.17	35.00	91.27	3.39	1.66	6.05	0.19	2.75	0.06	0.10
morus_alba	2.47	33.51	80.36	3.09	4.06	8.22	0.23	3.62	0.08	0.13
artocarpus_altilis	2.04	32.35	96.25	5.92	3.39	11.16	0.26	3.85	0.06	0.13
pteroceltis_tatarinowii	2.39	29.88	72.81	4.30	2.14	6.20	0.50	2.13	0.06	0.04
zelkova_serrata	1.70	39.50	39.45	3.12	4.23	4.16	0.16	1.85	0.04	0.13
celtis_bungeana	1.65	37.55	77.21	1.88	3.47	3.58	0.17	1.56	0.05	0.05
celtis_koraiersis	2.06	30.05	74.68	5.24	2.66	5.36	0.33	2.41	0.07	0.15
ulmus_lamellosa	2.01	32.48	86.07	5.49	2.15	5.36	0.20	4.37	0.06	0.10
ulmus_pumila	2.16	34.05	56.58	3.73	1.48	5.11	0.19	2.03	0.07	0.12
ulmus_macrocarpa	2.22	37.52	51.93	2.49	1.31	4.76	0.11	2.13	0.07	0.06
ulmus_parvifolia	2.37	34.94	58.66	4.71	3.72	6.01	0.29	2.69	0.08	0.16
