# Supplementary material for: Evolutionary Position and Leaf Toughness Control Chemical Transformation of Litter, and Drought Reinforces This Control: Evidence from a Common Garden Experiment across 48 Species
Source: PLoS One. 2015 Nov 17;10(11):e0143140. doi: 10.1371/journal.pone.0143140 (PMC4648592; doi:10.1371/journal.pone.0143140)
Supplement: S4 Table — (PDF) [file pone.0143140.s005.pdf]

S4 Table. Litter mass loss, fragmentation index and leaf toughness across 48 species

species	mass loss belowground	Fragmentation belowground	mass loss aboveground	Fragmentation aboveground	Leaf toughness
ginkgo_biloba	0.45	0.46	0.52	0.53	156.0807
magnolia_denudata	0.64	0.64	0.58	0.59	100.1633
platanus_acerifolia	0.36	0.38	0.42	0.42	89.4967
diospyros_kaki	0.43	0.46	0.33	0.34	114.9013
eucommia_ulmoides	0.37	0.40	0.58	0.63	59.156
tomentosa	0.52	0.56	0.62	0.70	79.8047
forsythia_suspensa	0.62	0.67	0.57	0.59	119.4753
fraxinus_mandschurica	0.61	0.64	0.55	0.57	86.794
syringa_oblata	0.60	0.63	0.71	0.72	112.2973
syringa_pekinensis	0.64	0.67	0.65	0.63	72.134
paeonia_suffruticosa	0.83	0.89	0.75	0.78	121.5093
lagerstroemia_indica	0.70	0.76	0.63	0.64	69.154
toxicodendron_vernicifluum	0.53	0.57	0.68	0.74	108.806
acer_truncatum	0.50	0.53	0.65	0.67	113.9573
ailanthus_altissima	0.67	0.73	0.63	0.65	86.9067
euonymus_maackii	0.71	0.78	0.74	0.79	113.0513
populus_tomentosa	0.49	0.52	0.58	0.59	115.2367
salix_matsudana	0.69	0.73	0.65	0.61	97.848
cercis_chinensis	0.53	0.56	0.42	0.43	112.138
sophora_japonica	0.63	0.68	0.70	0.72	86.6607
robinia_pseudoacacia	0.64	0.68	0.71	0.77	59.6187
juglans_regia	0.57	0.57	0.56	0.61	76.2073
quercus_aliena var.pekingensis	0.43	0.45	0.34	0.34	118.8213

quercus_aliena var.acuteserrata	0.41	0.42	0.37	0.38	107.846
quercus_acutissima	0.36	0.38	0.37	0.35	153.1527
amygdalus_davidiana	0.69	0.73	0.64	0.66	60.418
rosa_xanthina	0.69	0.77	0.73	0.80	57.6733
armeniaca_mume var.bungo	0.65	0.70	0.71	0.74	83.07
chaenomeles_speciosa	0.62	0.65	0.56	0.57	96.562
crataegus_pinnatifida	0.83	0.87	0.67	0.71	75.9913
prunus_sargentii	0.77	0.84	0.65	0.70	69.3933
prunus_yedoensis	0.65	0.71	0.61	0.65	77.6667
cerasus_glandulosa	0.61	0.64	0.70	0.74	51.0587
elaeagnus_pungens	0.37	0.37	0.62	0.80	70.3453
elaeagnus_umbellata	0.26	0.27	0.48	0.50	81.0913
rhamnus_davurica	0.92	0.95	0.91	0.93	77.326
ziziphus_jujuba var.spinosa	0.57	0.64	0.77	0.85	76.096
maclura_tricuspidata	0.67	0.76	0.78	0.82	103.0707
morus_alba	0.66	0.70	0.75	0.79	85.446
artocarpus_altilis	0.78	0.83	0.88	0.91	88.9727
pteroceltis_tatarinowii	0.73	0.78	0.85	0.86	56.36
zelkova_serrata	0.39	0.45	0.38	0.40	115.378
celtis_bungeana	0.45	0.50	0.57	0.60	114.7787
celtis_koraiersis	0.61	0.66	0.79	0.82	104.6593
ulmus_lamellosa	0.69	0.72	0.62	0.64	134.018
ulmus_pumila	0.57	0.63	0.68	0.72	73.646
ulmus_macrocarpa	0.56	0.58	0.45	0.46	80.1193
ulmus_parvifolia	0.66	0.68	0.72	0.76	117.1733
